# Surveillance and epidemiological characterization of human adenovirus infections among outpatient children with acute gastroenteritis during the COVID-19 epidemic in Shanghai, China

**DOI:** 10.1186/s12985-023-02105-z

**Published:** 2023-06-21

**Authors:** Lijuan Lu, Ran Jia, Huaqing Zhong, Shuohua Duan, Menghua Xu, Liyun Su, Lingfeng Cao, Jin Xu

**Affiliations:** 1 Department of Clinical Laboratory, Children’s Hospital of Fudan University, National Children’s Medical Center, 399 Wanyuan Road, Shanghai, 201102 China; 2 School of Laboratory Medicine and Life Sciences, Wenzhou Medical University, Wenzhou, China; 3 Shanghai Institute of Infectious Disease and Biosecurity, Fudan University, Shanghai, China

**Keywords:** Human adenovirus, Children, Outpatient, Acute gastroenteritis, COVID-19

## Abstract

**Background:**

Human adenovirus (HAdV) has been recognized as one of the common enteric viruses associated with acute gastroenteritis (AGE) in children. The aim of this study was carried out to illustrate the epidemiological characterization of HAdV Infections among children younger than 15 years in Shanghai during COVID-19.

**Methods:**

During May 2020 and April 2022, 1048 fecal samples were collected from children ≤ 15 years diagnosed with AGE in the Children’s Hospital of Fudan University. HAdV was identified by PCR and sequenced with specific primers. All the obtained sequences were analyzed by MEGA (version 6.0). Demographic information and clinical features data were also collected and analyzed.

**Results:**

In total, 97 (9.3%, 97/1048) samples were detected to be HAdV during May 2020 and April 2022. We found an atypical upsurge in HAdV infection in the year 2021 after a major suppression in the year 2020. Approximately 84.5% (82/97) of HAdV-infected children were aged 0–60 months. Among the 97 HAdV-positive samples, only two species and five genotypes were detected. HAdV-F (88.7%, 86/97) was the most prevalent species and HAdV-F41 (87.6%, 85/97) was the most common genotype. Diarrhea, vomiting, and fever were the main clinical manifestations in children infected with HAdV. The children aged from 0 to 12 months showed simpler patterns of clinical presentation than those of children older than 13 months.

**Conclusions:**

Our findings described the epidemiological changes of HAdV infection in children with AGE during the COVID-19, which further underscored the importance of continuous surveillance of HAdV at both local and global scales.

## Introduction

Acute gastroenteritis (AGE) is a major public health problem in people of all ages and poses a significant health risk to the very young, including infants and young children [1–3]. According to previous reports, the virus is the most common cause of acute gastroenteritis in people under 18 years of age [4, 5]. Rotavirus, norovirus (NoV), human adenovirus (HAdV) and human astrovirus (HAstV) have been recognized as the major causes of AGE in children [4]. In addition, the global burden of disease (GBD) study used mathematical modeling to estimate that HAdV is the second most common cause of death in children with AGE, after rotavirus [6].

HAdV belongs to the family Adenoviridae within the genus Mastadenovirus. HAdV is a medium-sized (70–100 nm), non-enveloped virus with an icosahedral nucleocapsid containing a double-stranded linear DNA genome of 34–45 kbp [7]. Based on pathogenicity and genetic characteristics, HAdV has been classified into seven species A to G and 113 different HAdV genotypes have been identified [8–10]. HAdV is a highly contagious pathogen that can infect a variety of organ systems, including the gastrointestinal tract, urinary tract, eye, upper respiratory tract, lower respiratory tract and other systems [8]. Different species have different tissue tropisms. Among the seven species, the HAdV F species, called enteric HAdV, which includes HAdV-F40 and HAdV-F41 genotypes, has been identified as the major AGE agent [11–14]. In addition, some other non-enteric HAdV species (HAdV A-E and G species), such as HAdV A, HAdV B, HAdV C and HAdV D, have been frequently identified in stool samples from patients with AGE [10–12, 14–17].

The severe acute respiratory syndrome coronavirus 2 (SARS-CoV-2) epidemic, known as coronavirus disease 2019 (COVID-19), spread rapidly around the world shortly after its breakout in late 2019 [18]. The COVID-19 pandemic caused extraordinary changes in community behavior at the population level and remarkably reduced the prevalence of infectious diseases such as respiratory pathogens [19–21]. However, fewer studies have been conducted on the epidemiological characteristics of HAdV in children with AGE in the context of COVID-19 [22]. Therefore, this paper reported changes in the epidemiological characteristics of HAdV infections in children under 15 years of age with AGE in Shanghai during the COVID-19 pandemic.

## Materials and methods

### Study population and stool sample collection

A total of 1048 stool samples were collected from outpatients with AGE at the Children's Hospital of Fudan University, Shanghai, China, between May 2020 and April 2022. AGE was defined as three or more loose, watery, thin stools with a pasty texture, or the presence of mucous stools per day, possibly accompanied by vomiting, abdominal pain, fever, and lasting < 2 weeks [15, 23]. Patient information was obtained from medical records, including demographic data and clinical symptoms. Informed consent was not required from participants' or patients' guardians, as the stool samples included were residual samples from routine detection. Ethical approval for this study was granted by the Ethical Review Committee of the Children's Hospital of Fudan University.

## Nucleic acid extraction, detection of HAdV, NoV and HAstV

Total nucleic acids were extracted from 200 µl of a 10% fecal supernatant using a Viral Ex-DNA/RNA kit (Xi'an TianLong Science and Technology Co., Ltd) and eluted in 80 µl of DEPC water according to the manufacturer's instructions. All extracted genomes were kept frozen at − 70 °C for further use. In this study, a partial nucleotide sequence of the conserved region of the hexon was amplified for HAdV detection. The primers were hexAA1885 (5′–GCCSCARTGGKCWTACATGCACATC–3′) and hexAA1913 (5′–CAGCACSCCICGRATGTCAAA–3′). Briefly, an initial denaturation step at 94 °C for 2 min, followed by 40 cycles of 94 °C for 30 s, 55 °C for 30 s, 72 °C for 30 s and a final extension step at 72 °C for 7 min. PCR products were electrophoresed in a 2.0% agarose gel using Super GelBlue (Shanghai BioScience Co., Ltd.) and visualized using an automated gel image analysis system (Shanghai Tanon Life Science Co. LTD). The expected size of the amplicon was 308 bp. Detection of NoV and HAstV was performed as in our previous studies [24].

### Nucleotide sequencing and phylogenetic analysis of HAdV

All amplification products from HAdV-positive samples were subjected to nucleotide sequencing using the Sanger method by Sangon Biotech (Shanghai) Co., Ltd. Sequence alignment was performed in MEGA 6.0. A phylogenetic tree was constructed based on the nucleotide sequences of the hexon gene of HAdVs and reference sequences from GenBank. Phylogenetic analysis was performed using the Kimura 2-parameter model and maximum likelihood method in MEGA 6.0. The reliability of the tree was assessed by 1000 bootstrap replicates.

### Statistical analysis of major parameters

Statistically significant differences in detection rates and proportion of categorical variables were tested using Fisher's exact test, two-tailed chi-square test or corrected chi-square test in SPSS software, version 20 (IBM Corp., Armonk, NY, USA). *P* values < 0.05 were considered statistically significant.

## Results

### Demographic characteristics of the study population

From May 2020 to April 2022, 1048 cases of outpatient children ≤ 15 years with AGE were recruited for the current study. Among the patients, 60.3% (632/1048) were males and 39.7% (416/1048) were females. The age of the children ranged from 21 days to 15 years. The patients were divided into nine age groups including 0–1, 2–6, 7–12, 13–24, 25–36, 37–48, 49–60, 61–72 and > 73 months.

### Detection of HAdV in children with age

**Fig. 1:**
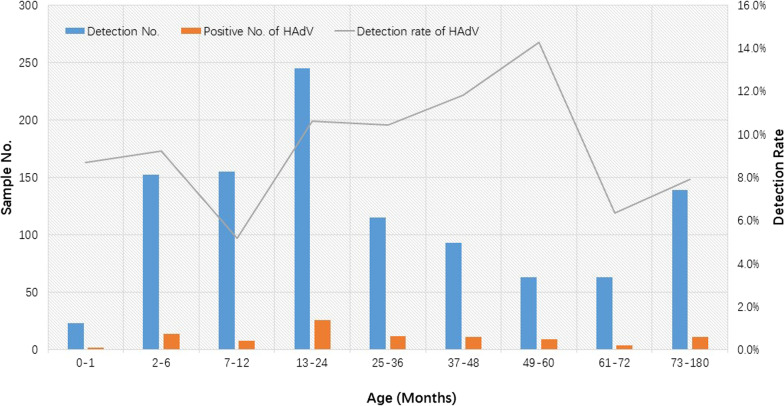
Age distribution of HAdV infections in Children with AGE in Shanghai during COVID-19

Of the 1048 stool samples, HAdV was detected in a total of 97 (9.3%, 97/1048) samples. HAdV broke out with an atypical upsurge of HAdV infection in the winter of 2020/2021 after a major suppression in early 2020 in Shanghai. Compared to 2020 (2.2%, 5/226), the detection rate of HAdV was significantly increased in 2021 (12.1%, 88/725) (χ^2^ = 19.238,* P* = 0.000). The positive rate for HAdV was 9.5% (60/632) in males and 8.9% (37/416) in females (χ^2^ = 0.107, *P* = 0.416). Furthermore, HAdV infections were detected in all nine age groups. Of the 97 positive samples, approximately 84.5% (82/97) of HAdV-infected children were aged 0–60 months. The detection rate of HAdV in children ≤ 60 months (9.7%, 82/846) and those aged 61 months to 15 years (7.4%, 15/202) were comparable (χ^2^ = 0.998, *P* = 0.348). Furthermore, the highest prevalence of HAdV infection was found in children aged 49–60 months (14.3%, 9/63) and the lowest detection rate of HAdV was found in children aged 7–12 months (5.2%, 8/155). Of note, two newborns diagnosed with AGE in this study were infected with HAdV (Fig. 1).

### Seasonality trends of HAdV infections

**Fig. 2:**
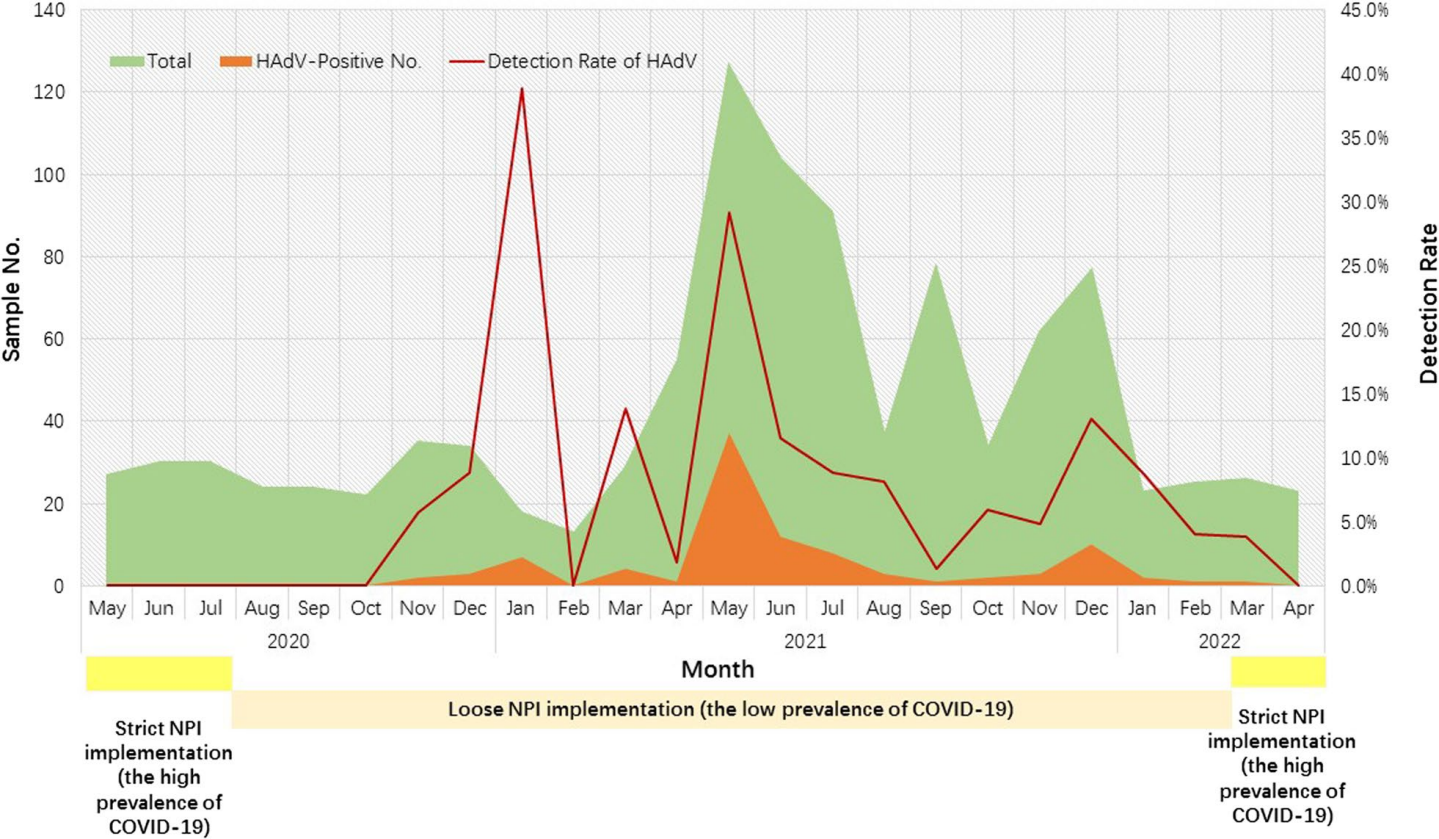
Seasonal trends of HAdV infections in Children with AGE in Shanghai during COVID-19 (strict NPI implementation with the high prevalence of COVID-19 indicates strategies including community lockdown, closure of schools and day-care centers; and loose NPI implementation with the low prevalence of COVID-19 indicates measures without community lockdown, closure of schools and day-care centers)

During the study period, HAdV showed an abnormal transmission pattern during the COVID-19 pandemic. HAdV infection was not detected from May to October 2020 but increased sharply in the winter month (January) 2021 (38.9%, 7/18). Then, from March 2021 to March 2022, HAdV infections were detected throughout the year, with a peak prevalence in early summer (May) 2021 (29.1%, 37/127). After that, the HAdV positive rate reached a small prevalence peak in the winter month (December) 2021 (13.0%, 10/77) (Fig. 2).

### HAdV species and genotypes

**Fig. 3:**
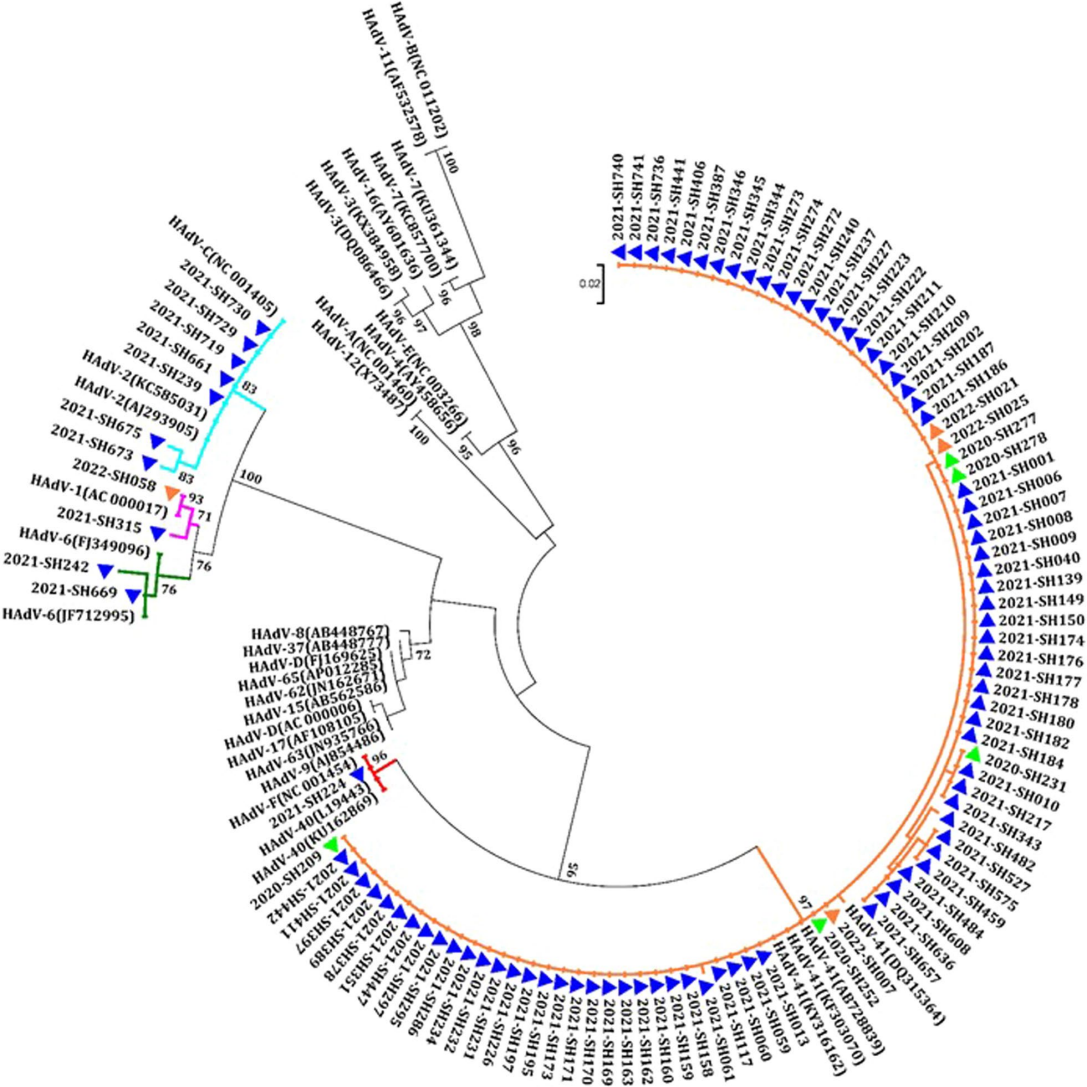
Phylogenetic analysis on the partial nucleotide sequences of the conserved region of HAdV detected in children with AGE during COVID-19. Green filled triangle: 2020, blue filled triangle: 2021, orange filled triangle: 2022

All 97 PCR-positive products were sequenced and phylogenetically analyzed. A phylogenetic tree based on the partial hexon nucleotide sequence (308 bp) was constructed to identify HAdV species and genotypes (Fig. 3). Two species, HAdV C and HAdV F were detected and five different genotypes were identified. Among them, HAdV F (88.7%, 86/97) was the most common species. Among the five different genotypes detected, HAdV-F41 (87.6%, 85/97) was the most common genotype, followed by HAdV-C2 (7.2%, 7/97), HAdV-C1, C6 (2.1%, 2/97) and HAdV-F40 (1.0%, 1/97) (Table 1). Furthermore, all five different genotypes were detected in 2021, whereas only one (HAdV-F41) and two genotypes (HAdV-C1 and HAdV-F41) were detected in 2020 and 2022, respectively.

**Table 1:** Numbers and constituent ratio of different species and genotypes of HAdV identified in this study

Species and genotypes	Number (% of total)
*HAdV-C*	
HAdV-1	2 (2.1)
HAdV-2	7 (7.2)
HAdV-6	2 (2.1)
*HAdV-F*	
HAdV-40	1 (1.0)
HAdV-41	85 (87.6)
Total	97 (100.0)

### Clinical features of children infected with HAdV

**Fig. 4:**
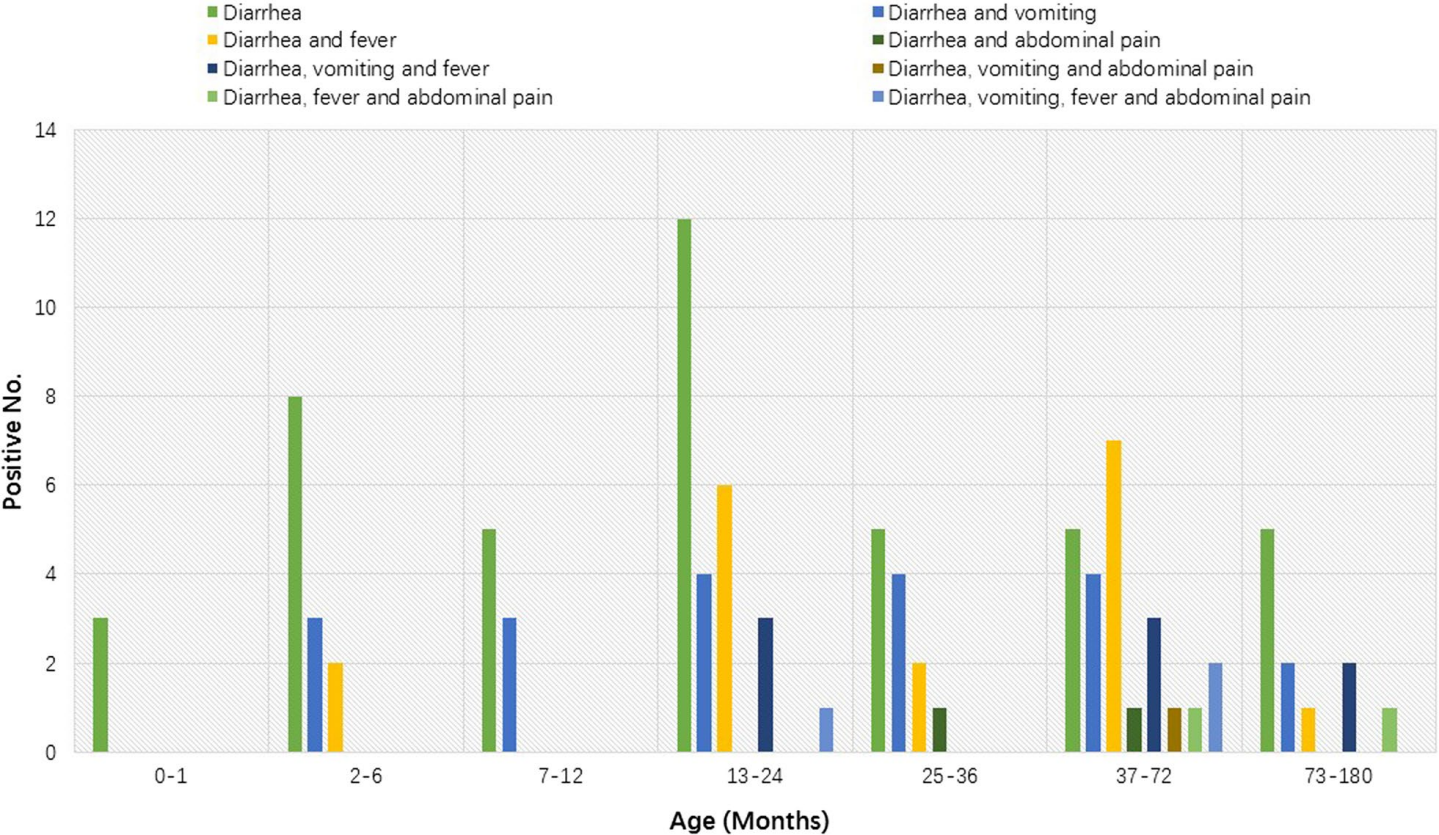
Clinical features of children with HAdV infections at different age groups

Regarding clinical features, we found that diarrhea, vomiting, fever and abdominal pain were common clinical manifestations in children infected with HAdV. The most frequent clinical feature of HAdV-infected children was diarrhea (100.0%, 97/97), followed by vomiting (33.0%, 32/97), fever (30.9%, 30/97) and abdominal pain (8.2%, 8/97). Among those children, about 45.4% (44/97) had diarrhea only, followed by children with diarrhea and vomiting (20.6%, 20/97) and children with diarrhea and fever (17.5%, 17/97) (Table 2). The children aged from 0 to 12 months showed simpler patterns of clinical presentation than those of children older than 13 months (Fig. 4). In addition, children infected with non-enteric HAdV were more likely to develop fever than those infected with enteric HAdV (χ^2^ = 5.931, *P* = 0.023).

**Table 2:** Clinical features observed among children infected with enteric and non-enteric HAdV

Clinical symptoms	Enteric HAdV n (m)	Non-enteric HAdV n (m)	Total n (m)
Diarrhea	42 (48.3)	3 (27.3)	44 (45.4)
Diarrhea and vomiting	20 (23.0)	–	20 (20.6)
Diarrhea and fever*	11 (12.6)	6 (54.5)	17 (17.5)
Diarrhea and abdominal pain	2 (2.3)	–	2 (2.1)
Diarrhea, vomiting and fever	7 (8.0)	1 (9.1)	8 (8.2)
Diarrhea, vomiting and abdominal pain	1 (1.1)	–	1 (1.0)
Diarrhea, fever and abdominal pain	1 (1.1)	1 (9.1)	2 (2.1)
Diarrhea, vomiting, fever and abdominal pain	3 (3.4)	–	3 (3.1)
Total	86 (100.0)	11 (100.0)	97 (100.0)

*P < 0.05;* n*: HAdV-positive number;* m*: component ratio of different clinical presentations in children infected with HAdV

### Co-infection of HAdV with NoV and HAstV

Among the 97 HAdV-positive samples, single infections were detected in 81.4% (79/97) of the HAdV-positive samples, whereas mixed infections were detected in 18.6% (18/97) of the same samples. Of the 18 mixed infections, the most common dual infection was HAdV and NoV (72.2%, 13/18), followed by HAdV and HAstV (27.8%, 5/18). Mixed infections detected in males (61.6%, 11/18) and females (38.9%, 7/18) were similar (χ^2^ = 1.778, *P* = 0.159).

## Discussion

HAdV is one of the important pathogens causing AGE in children. There is an urgent need for comprehensive surveillance to better understand the epidemiological characteristics of HAdV in children with AGE in Shanghai during the COVID-19 pandemic. Therefore, we aimed to comprehensively evaluate the epidemiology of HAdV infections in children with AGE in Shanghai from May 2020 to April 2022.

During the study, the overall HAdV detection rate in children with AGE was 9.3%, which was significantly higher than our previous data in Shanghai (3.5%) (2017–2018) before the COVID-19 pandemic [25]. According to the detailed monthly distribution of HAdV infections, HAdV infections tended to occur in oscillatory fluctuations. None of the HAdV infections were detected from May to October 2020 due to the restrictive non-pharmaceutical interventions (NPI) were applied, such as lockdowns, closure of schools and day-care centers, social distancing, increased hygiene awareness and wearing of masks against COVID-19 in the preliminary stage of the COVID-19 epidemic. A similar phenomenon was reported in Brazil from April to September 2020 [22]. In addition, other studies also reported a decrease in the incidence of various infectious diseases of viral etiology in children during the COVID-19 pandemic, including enteric viruses [26–30]. All these studies reflected that the NPI strictly implemented during the COVID-19 pandemic played an important role in reducing viral infections including gastroenteritis viruses in children. In contrast, the normalization of life created conditions for the prevalence of HAdV among children with AGE in 2021 with the low prevalence of COVID-19. HAdV infections showed a gradual increase, peaking in January 2021 (38.9%) with the gradual reopening of the community in Shanghai [19]. Thereafter, the HAdV detection rate reached the second and third highest percentage positive rate (29.1% in May and 13.0% in December) throughout the study in 2021, followed by a distinct decrease in March 2022 because of the lockdown in Shanghai. These data suggested that the prevalence of HAdV showed an abnormal transmission pattern during the COVID-19 pandemic in Shanghai.

In addition, we also compared the distribution of HAdV infection in children of different genders and ages. Our results suggest that gender did not play a role in HAdV infection during this period, which is consistent with findings described in our previous study and worldwide before the COVID-19 epidemic [11, 12, 25, 31, 32]. Regarding age groups, a higher HAdV positivity rate was observed in children ≤ 60 months than in children > 61 months, reinforcing the global observation that HAdV is an important pathogen in childhood diarrhea. However, children aged 49–60 months were the most susceptible group to HAdV infection in this study, whereas HAdV infection was most frequently detected within 37–48 months in our previous study before COVID-19 [25]. We speculated that this may be related to older children having much greater opportunities to be exposed to and infected with HAdV due to increased outdoor activities. It is worth mentioning that two neonates with AGE were infected with HAdV, which reminded us to pay attention to HAdV infections in this age group.

According to our data, the prevalence of HAdV in children was more frequent in the cold season, which was similar to our previous study in outpatients (2012–2016) and Shandong Province, China (2017–2018) [13, 33]. Furthermore, an unusual prevalence peak was also identified in May 2021. However, the seasonal distribution of HAdV infection remains a controversial issue worldwide [11, 14, 15, 34–36]. For example, the pattern of HAdV infection in Shanghai in 2017–2018 among children with AGE was not observed as a consistent seasonal pattern similar to Brazil [25, 37, 38]. In Thailand (2011–2017) and India (2013–2014), the most prevalent HAdV infections occurred during the rainy season [11, 35]. We speculated that the heterogeneity in the seasonal distribution of HAdV infection could be due to different epidemiological scenarios, weather patterns, differences in population immunity factors and major public health events such as COVID-19.

HAdV is recognized as the common pathogen associated with acute gastroenteritis, especially enteric HAdV. During the period of this study, five different HAdV genotypes belonging to two different species were identified. In line with worldwide findings, enteric HAdV infections were the leading species of positive cases detected, with a predominance of HAdV-F41 in this study [17, 39, 40]. However, the prevalence of another enteric HAdV genotype F40 rarer compared with HAdV-F41. These results were similar to previous studies in Hangzhou (2017–2018) and Shanghai (2012–2018) in China and Brazil (2012–2017) [25, 33, 40]. Conversely, HAdV-F40 was detected more frequently than HAdV-F41 in India (2013–2014) [35]. In contrast to previous studies in Shanghai (2012–2018), species C was the only non-enteric HAdV species detected during the COVID-19 epidemic [33, 38]. HAdV-C2 was the most frequently detected non-enteric HAdV, which was consistent with findings from Brazil (2012–2017) and Shanghai (2017–2018) [25, 37]. Furthermore, previous studies conducted in some developing countries have reported results for non-enteric HAdV, with type C being the most prevalent and type A, B and D alternating as the second most prevalent [11, 12]. However, it remains controversial whether diarrhea is directly caused by the non-enteric HAdV detected in children with AGE. Therefore, more work is needed to establish this association in the future.

In this study, we also tested for other enteric viruses, including NoV and HAstV. We found that 18.6% of HAdV-positive samples were co-infected with these two viruses, while 81.4% of cases were infected with HAdV alone. Co-infection of HAdV with other enteric viruses is commonly reported and studies from Brazil, Bangladesh, USA and France have demonstrated co-infection of HAdV with rotavirus and NoV, but at lower rates [17, 24, 34, 41–43]. However, we could not exclude the possibility that some AGE cases were caused by bacteria or other pathogens not investigated in the present study.

We also analyzed the clinical characteristics of the HAdV-positive children. According to the medical history of the HAdV-positive children, the most typical clinical symptoms were diarrhea, followed by vomiting, fever and abdominal pain. This finding was consistent with previous reports of HAdV-infected children before the COVID-19 epidemic. In addition, children infected with non-enteric HAdV are more likely to develop fever than those infected with enteric HAdV. In addition, younger children infected with HAdV in this study had milder clinical symptoms.

## Conclusion

In conclusion, the current study is the first to comprehensively report the epidemiological characteristics of HAdV among children with AGE aged 0–15 years in Shanghai during COVID-19. It is clear that COVID-19 affected the transmission pattern of HAdV during the COVID-19 period. Our results suggest that HAdV-F41 is still the major cause of AGE in children ≤ 5 years of age. These findings are a timely warning to us that HAdV infections still deserve full attention to avoid unusual rebounds and outbreaks.

## Data Availability

All data generated during the current study are available upon request by contacting the corresponding authors.

## Abbreviations

HAdV: Human adenovurus
NoV: Norovirus
HAstV: Human astrovirus
AGE: Acute gastroenteritis
COVID-19: Coronavirus disease 2019
GBD: Global burden of disease
SARS-CoV-2: Severe acute respiratory syndrome coronavirus 2
